# Does low pre-dialysis blood volume increase survival? A call for caution

**DOI:** 10.1177/03913988251351523

**Published:** 2025-06-19

**Authors:** Simon Krenn, Daniel Schneditz, David Keane, Sebastian Mussnig, Manfred Hecking

**Affiliations:** 1 Center for Public Health, Department of Epidemiology, Medical University of Vienna, Vienna, Austria; 2 Otto Loewi Research Center for Vascular Biology, Immunology and Inflammation, Division of Physiology and Pathophysiology, Medical University of Graz, Graz, Austria; 3 CÚRAM Research Ireland Centre for Medical Devices, University of Galway, Galway, Ireland; 4 Lilibeth Caberto Kidney Clinical Research Unit, London Health Sciences Center Research Institute, London, Ontario, Canada; 5 Department of Medicine III, Division for Nephrology and Dialysis, Medical University of Vienna, Vienna, Austria; 6 KfH-Nierenzentrum Weiden, KfH Kuratorium für Dialyse und Nierentransplantation e.V., Weiden, Germany

In their recent article entitled “Absolute blood volume and long-term survival in chronic hemodialysis patients,” Kron et al.^1^ investigated the relationship between pre-dialysis specific blood volume (SBV) and 5-year survival of patients on hemodialysis. They found much higher survival rates below an SBV threshold of 75 mL/kg at treatment start (defined as euvolemia), especially in patients with mildly impaired left-ventricular ejection fraction (LVEF) 40%–59%. We commend the authors’ initiative to perform a survival analysis based on SBV. However, we feel obliged to raise several concerns regarding (1) physiological assumptions, (2) methodology, and (3) clinical recommendations.

(1) Physiological assumptions: There is presently no evidence suggesting that 75 mL/kg SBV constitutes a threshold for euvolemia. SBV is known to be systematically lower at higher BMI.^2,3^ High BMI is further known to be associated with a survival benefit in populations on hemodialysis (known as reverse epidemiology^4^). In the present study, the high-survival group with SBV <75 mL/kg also had significantly higher BMI (28.65 kg/m^2^ vs 26.18 kg/m^2^), potentially confounding the results in favor of low SBV.^1^ Hypovolemia and its risks on the other hand were ignored, although the authors previously reported that patients with SBV <65 mL/kg more frequently experienced intradialytic morbid events.^5^
(2) Methodology: Since this was an ancillary, post hoc analysis of a previously published study, several problems arise. Reuse of data, non-prespecified analyses, and small sample sizes limit the reliability of findings. Prevalent patients with varying dialysis vintage were included, introducing potential immortal time bias. Adjustment for relevant covariates (like BMI, age, and vintage) was omitted and—judging by Kaplan-Meier plots—survival data had only annual resolution. Inclusion timeline, censoring timepoints, and the causes of death were not described. For the LVEF sub-analysis (*n* = 19), distribution of patient characteristics was also not disclosed.
(3) Clinical recommendations: The authors concluded that results “might confirm the integration of absolute blood volume measurement in volume management strategies” and “demonstrate the significance of strict volume control, particularly in cases of reduced cardiac function,” although patients with volume expansion >4 L, NYHA class >II, and left-ventricular ejection fraction (LVEF) <40% were excluded. They elaborated that if “hemodialysis patients with cardiac dysfunction are maintained in a euvolemic status, their mortality is not increased” and proposed to “inform our patients in detail about these quite optimistic findings”. Given the broad exclusion criteria, reuse of data, lack of adjustment, arbitrary time zero, low number of patients, confounding by BMI, and the fact that neither maintenance of euvolemic status nor specific measures of volume control (nor indeed any other intervention) were performed in this post hoc analysis, we recommend readers to use discretion in evaluating these statements.

In summary, while we appreciate and thank the authors for their hard work and their commitment to this field of research, we hope to have provided readers with some nuance in the study’s interpretation. In the absence of adequately powered, prospective interventional trials, we suggest the present SBV cutoffs should not be used as targets, especially not in patients with low BMI who might be overtreated due to their naturally higher SBV.
